# Preoperative Mean Platelet Volume and Platelet Distribution Width Predict Postoperative Sepsis in Patients with Colorectal Cancer

**DOI:** 10.1155/2019/9438750

**Published:** 2019-10-30

**Authors:** Xue-ting Li, Zibo Yan, Rui-tao Wang, Kai-jiang Yu

**Affiliations:** ^1^Department of Intensive Care Unit, Harbin Medical University Cancer Hospital, Harbin Medical University, Harbin, Heilongjiang 150081, China; ^2^Department of Planed Immunization, Heilongjiang Provincial Center for Disease Control and Prevention, Harbin, Heilongjiang 150030, China; ^3^Department of Internal Medicine, Harbin Medical University Cancer Hospital, Harbin Medical University, Harbin, Heilongjiang 150081, China; ^4^Institute of Intensive Care Unit, Heilongjiang Academy of Medical Science, Harbin, Heilongjiang 150081, China; ^5^Department of Intensive Care Unit, The First Affiliated Hospital of Harbin Medical University, Harbin Medical University, Harbin, Heilongjiang 150081, China

## Abstract

**Purpose:**

Mean platelet volume (MPV) and platelet distribution width (PDW) have been used to reflect the platelet activity in clinics. We assessed initial serum MPV and PDW levels in colorectal cancer (CRC) patients, in predicting the development of sepsis in CRC patients postoperatively.

**Patients and Methods:**

This study included 220 patients diagnosed with CRC. 55 patients were stratified to one group that developed sepsis postoperatively, and 165 patients were stratified to the other group that did not develop sepsis postoperatively. Clinical and laboratory characteristics were collected 3 days before the operation.

**Results:**

MPV (*p* < 0.001) was significantly higher and PDW (*p* < 0.001) was significantly lower in the sepsis group than in the nonsepsis group. Either MPV or PDW is independently associated with ICU mortality in sepsis patients with CRC. MPV is independently associated with 14-day, 28-day, and 90-day mortality and PDW is independently associated with 90-day mortality in patients with CRC. The prevalence of sepsis increased as MPV tertiles increased (*p* < 0.001), and the prevalence of sepsis increased as PDW tertiles decreased (*p* < 0.001).

**Conclusions:**

Serum MPV and PDW levels between CRC patients with/without sepsis postoperatively are significantly different. The initial serum MPV or PDW levels can potentially serve as a predictor of sepsis in CRC patients postoperatively.

## 1. Introduction

Cancer is the leading cause of high mortality worldwide and causes heavy socioeconomic impact [1–3]. Among cancer patients, death due to sepsis-related multiorgan failure is more frequent than death due to cancer itself [4–7]. Colorectal cancer (CRC) is one of the commonest malignant diseases in China and is a frequent cause of cancer-related death [8].

Sepsis is identified as life-threatening organ dysfunction caused by a dysregulated host response to infection [9]. The multiple organ failure caused by sepsis is the most lethal cause of surgery in ICU [10]. A study reported the mortality rate for cancer patients with septic shock admitted to the critical care unit (ICU) is nearly 54% [11].

It is well known that platelets are related to the hemostasis and coagulation function [12]. In recent years, more and more studies have confirmed that activated platelets are involved in the development and metastasis of tumors [13, 14]. Interaction between platelets and the tumor cells is not depending on the platelet quantity but on the volume and size because larger platelets have more granules and receptors [15]. Platelet volume indices have been used to reflect the platelet activity in clinics, including mean platelet volume (MPV) and platelet distribution width (PDW) [16]. MPV is found as an indicator of activated platelets and associated with many cancers [17–19]. PDW, another platelet parameter, indicates variation in platelet size and differentially diagnoses thrombocytopenia [16].

Even though more and more precaution was paid to hospitalized patients, sepsis incidence in cancer patients after surgery still takes up a significant burden of illness [20]. Patients undergoing surgery for CRC are at particular risk of sepsis because of underlying malignancy, being immunocompromised associated with cancer management and the complexity of surgical procedures performed [21]. Therefore, it is urgent to find out biomarkers which can predict the occurrence of postoperative sepsis in CRC patients. This study aimed to address the role of MPV and PDW as biomarkers in predicting the development of postoperative sepsis in CRC patients.

## 2. Patients and Methods

### 2.1. Study Design

This was a prospective study. The study protocol was approved by the Institutional Review Board of the Harbin Medical University Cancer Hospital. All patients signed informed consent.

### 2.2. Patients and Data Collection

CRC patients were admitted to the Harbin Medical University Cancer Hospital for surgery between January 1, 2015, and December 30, 2017. All patients underwent complete surgical resection, and the pathologic diagnoses were histologically confirmed by two experienced pathologists. There were 55 sepsis cases after surgery (mean age 63.4 ± 9.7 years, range 57–86 years). Using incidence density sampling, we matched the 55 sepsis patients with 165 controls by age, sex, and BMI (mean age 63.4 ± 9.0 years, range 46–78 years). All sepsis patients fulfilled the criteria of sepsis (SEPSIS-3) [22]. Cases were included if they met the following criteria: (1) age > 18 years; (2) patients who underwent complete surgical resection and diagnosis of CRC cancers being confirmed by histology; (3) diagnosis of sepsis after surgery in the intensive care unit (ICU); and (4) patients who did not receive preoperative chemotherapy or radiation therapy. Exclusion criteria included HIV infection, neutropenia (<500 neutrophils/mm^3^), and the medical treatment with steroids. The Acute Physiology and Chronic Health Evaluation (APACHE) II scores and the Sequential Organ Failure Assessment (SOFA) scores were determined.

### 2.3. Clinical Examination and Biochemical Measurements

All subjects underwent physical examination. Body mass index (BMI) was calculated as the ratio of weight (kg) to height squared (m^2^). Clinical data including medical history and medication use were recorded for each subject. Venous blood samples were collected 3 days before surgery. White blood cell (WBC), hemoglobin, and platelet indices were measured by an auto analyzer (Sysmex XE-2100, Kobe, Japan). The normal ranges of MPV and PDW in our hospital are 7–11 fL and 11–17%, respectively. The inter- and intra-assay coefficients of variation (CVs) of all these assays were below 5%.

### 2.4. Outcomes

The primary outcome of this study was to assess the predicting role of MPV and PDW in CRC patients who will be subjected to sepsis. Secondary outcomes were to certify if MPV or PDW can be a biomarker to forecast the ICU mortality or 28-day and 90-day mortality in sepsis patients with CRC.

### 2.5. Statistical Analysis

All statistical analyses were performed using SPSS Statistics version 22.0 (SPSS Inc., Chicago, IL, USA). The descriptive statistics are presented as means ± SD or medians (interquartile range) for continuous variables and percentages of the number for categorical variables. When baseline characteristics between two groups were compared, normally distributed continuous variables were compared with Student's *t*-test and skewed-distributed with the Mann–Whitney *U* test. The chi-square test was used for categorical variables. The odds ratios (ORs) and 95% confidence intervals (95% CIs) for sepsis were calculated using conditional logistic regression analysis. *p* < 0.05 was considered to indicate a statistically significant difference.

## 3. Results

The study cohort included a nonsepsis group (*n*=165) and a sepsis group (*n*=55). The nonsepsis group included 99 men and 66 women. The sepsis group included 33 men and 22 women. Their characteristics are shown in Table 1; the mean age in the sepsis group is 63.4 ± 9.7 years, compared with the mean age of 63.4 ± 9.0 years in the nonsepsis group. There was no significant difference between the sepsis group and the nonsepsis group with regard to age, gender, body mass index (BMI), smoking, drinking, WBC, platelet count, creatinine, ALP, fibrinogen, CEA, and comorbidities. It was observed that hemoglobin, albumin, and PDW were significantly decreased in the sepsis group than in the nonsepsis group. However, MPV, LDH, APACHE II score, SOFA score, length of ICU stay, and ICU mortality were significantly increased in the sepsis group than in the nonsepsis group.

Conditional logistic regression analysis showed that albumin (HR, 0.883; CI, 0.829–0.940; *p* < 0.001), MPV (HR, 2.125; CI, 1.677–2.694; *p* < 0.001), and PDW (HR, 0.756; CI, 0.645–0.886; *p* < 0.001) were independently associated with postoperative sepsis in CRC patients, as shown in Table 2. In addition, hemoglobin (HR, 1.029; CI, 1.002–1.057; *p*=0.038), albumin (HR, 0.884; CI, 0.805–0.970; *p*=0.01), MPV (HR, 1.841; CI, 1.369–2.476; *p* < 0.001), and PDW (HR, 0.669; CI, 0.537–0.833; *p* < 0.001) were independently associated with ICU mortality in sepsis patients with CRC, as shown in Table 3. Moreover, it is shown in Table 4 that albumin (HR, 0.842; CI, 0.772–0.917; *p* < 0.001) and MPV (HR, 0.372; CI, 1.042–1.807; *p*=0.024) were independently associated with 28-day mortality. Albumin (HR, 0.867; CI, 0.809–0.929; *p* < 0.001), MPV (HR, 1.626; CI, 1.309–2.019; *p* < 0.001), and PDW (HR, 0.830; CI, 0.710–0.970; *p*=0.019) were independently associated with 90-day mortality in patients with CRC.

**Figure 1:**
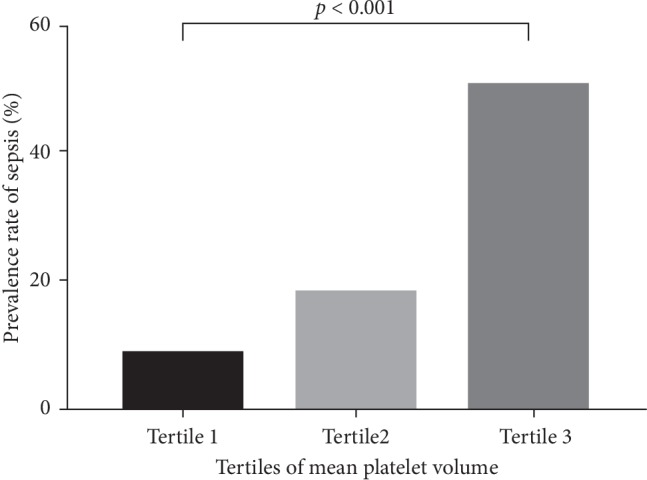
Association between MPV levels and the prevalence rate of sepsis (%). Participants were stratified into tertiles according to their MPV levels. Tertile 1 (T1) was MPV ≤ 8.0 fL, tertile 2 (T2) was MPV 8.1–9.0 fL, and tertile 3 (T3) was MPV ≥ 9.1 fL. The prevalence rate of sepsis in T1, T2, and T3 was 8.75%, 18.31%, and 50.72%, respectively.

The association between MPV levels and the prevalence rate of sepsis (%) is shown in Figure 1. All the 220 participants (nonsepsis group + sepsis group) were stratified into tertiles according to their MPV levels. Tertile 1 (T1) was MPV ≤ 8.0 fL, tertile 2 (T2) was MPV 8.1–9.0 fL, and tertile 3 (T3) was MPV ≥ 9.1 fL. The prevalence rate of sepsis in T1, T2, and T3 was 8.75%, 18.31%, and 50.72%, respectively.

**Figure 2:**
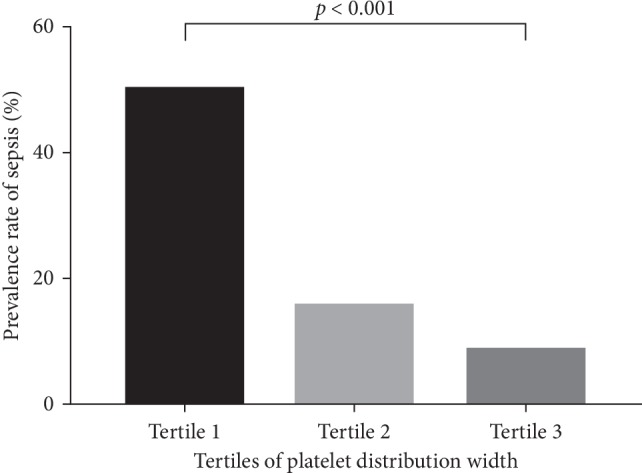
Association between PDW levels and the prevalence rate of sepsis (%). Participants were stratified into tertiles according to their PDW levels. Tertile 1 (T1) was PDW ≤ 16.5%, tertile 2 (T2) was PDW 16.6–17.4%, and tertile 3 (T3) was PDW ≥ 17.5%. The prevalence rate of sepsis in T1, T2, and T3 was 50.00%, 15.79%, and 8.57%, respectively.

The association between PDW levels and the prevalence rate of sepsis (%) is shown in Figure 2. Participants were stratified into tertiles according to their PDW levels. Tertile 1 (T1) was PDW ≤ 16.5%, tertile 2 (T2) was PDW 16.6–17.4%, and tertile 3 (T3) was PDW ≥ 17.5%. The prevalence rate of sepsis in T1, T2, and T3 was 50.00%, 15.79%, and 8.57%, respectively.

## 4. Discussion

In this study, we found that elevated MPV levels or reduced PDW levels before surgery are associated with postoperative sepsis events. In addition, MPV or PDW was independently associated with ICU mortality in CRC patients. Moreover, MPV was independently associated with 14-day mortality, 28-day mortality, and 90-day mortality in CRC patients; meanwhile, PDW was independently associated with 90-day mortality in CRC patients.

The mechanisms to explain the association between preoperative MPV or PDW and postoperative sepsis events remain unclear. The MPV is on behalf of an average size of platelets. Increased MPV levels are involved in the mechanism of platelet activation [23]. PDW reflected the heterogeneity in platelet size. A high value of PDW suggests a large range of platelet size due to swelling, destruction, and immaturity [24]. PDW and MPV are common indices which are associated with platelet activation and function [25]. In our study, we observed the patients who developed postoperative sepsis have higher preoperative MPV levels but lower PDW levels. This is maybe due to the cancer-associated inflammation. Studies have shown that the original chronic inflammatory disease directly causes at least 20% of cancers, especially in colorectal cancer [26, 27]. More cytokines are generated during the inflammation course in cancer, such as IL-6 and IL-11, and highly upregulated in many cancers [28]. In addition, previous research confirmed that tumors could promote platelet production and activation through IL-6 signaling [29]. TLR2 is a toll-like receptor protein which is expressed on megakaryocytes and platelets and is involved in innate immune responses. Interactively, inflammation mediated platelet activation through cytokines [30].

Consistent with Li et al.'s study, elevated MPV levels in CRC patients predicted the worse outcomes [31]. One clinical study uncovered a phenomenon where there were increased MPV levels and the thrombocyte consumption in infections [32]. Cekmez et al. showed that the MPV levels have positive relation with the incidence of early sepsis [33]. A study of neonatal sepsis confirmed that patients with neonatal sepsis had high levels of MPV [34]. Orak et al. found high MPV levels were significant in terms of prognosis and mortality in sepsis patients [32]. In our study, the elevated MPV levels were associated with ICU mortality in CRC patients similar to those reported in other studies. Furthermore, we had firstly confirmed the predictive effect of preoperative MPV levels on postoperative patients with sepsis. However, Zhu et al. reported the change in PDW in colorectal cancer patients was significantly higher versus that in both colorectal adenoma patients and healthy participants [35]. Our conflicting data are maybe due to sepsis, small sample sizes, failure to exclude confounding factors, different tumor types, and selected populations. Therefore, further research is needed for PDW.

Our study has a few limitations: Firstly, our study is single-centered, and there were only 220 patients included. In addition, this study lacks mechanistic data to explain the role of MPV and PDW in the development of sepsis. Moreover, we only assessed the initial MPV and PDW levels, and we had no serial data. Time-dependent changes in MPV and PDW levels would be more valuable.

## 5. Conclusions

In conclusion, either increased MPV or decreased PDW is an independent predictor of postoperative sepsis after CRC surgery. Our data shed light on the value of MPV and PDW in sepsis prevention.

## Figures and Tables

**Table 1:** Baseline characteristics of CRC patients stratified by development of sepsis status after operation.

Variables	Sepsis, *n*=55	Nonsepsis, *n*=165	*p* value
Age (years)	63.4 ± 9.7	63.4 ± 9.0	0.993
Gender (male, %)	33 (60.0)	99 (60.0)	1.000
BMI (kg/m^2^)	23.5 ± 3.1	23.5 ± 3.3	0.927
Smoker (%)	9 (16.4)	33 (20.0)	0.552
Drinking (%)	8 (14.5)	35 (21.2)	0.280
WBC (×10^9^/L)	8.58 ± 6.08	7.15 ± 2.63	0.093
Hemoglobin (g/L)	113.4 ± 26.7	130.6 ± 24.6	<0.001
Platelet count (×10^9^/L)	272.5 ± 131.8	272.5 ± 84.4	0.999
MPV (fL)	9.6 ± 1.3	8.4 ± 1.7	<0.001
PDW (%)	14.6 ± 2.8	17.2 ± 1.1	<0.001
Creatinine (umol/L)	84.1 ± 37.4	88.0 ± 20.9	0.460
Albumin (g/L)	35.0 ± 6.9	44.7 ± 6.8	<0.001
LDH (U/L)	166 (146–193)	145 (125.5–179)	0.003
ALP (U/L)	83.0 (68.0–108.0)	87.0 (75.0–109.0)	0.196
Fibrinogen (g/L)	3.44 ± 1.02	3.69 ± 1.03	0.126
CEA (ng/ml)	5.16 (2.19–11.64)	4.72 (2.26–15.78)	0.779
Comorbidities, *n* (%)			
Coronary artery disease	10 (18.2)	33 (20.0)	0.768
COPD	0	0	
Hypertension	12 (21.8)	27 (16.4)	0.359
Diabetes mellitus	10 (18.2)	31 (18.8)	0.920
APACHE II score	17 (12–22)	13 (11–14)	<0.001
SOFA score	9 (6–10)	6 (6–8)	0.005
Length of ICU stay, median (IQR)	4 (1–7)	1 (1–1)	<0.001
ICU mortality (%)	15 (27.3)	0 (0)	<0.001
Tumor location			0.392
Colon	31 (56.4)	82 (49.7)	
Rectum	24 (43.6)	83 (50.3)	
Tumor size (cm)			0.576
<5.0	32 (58.2)	103 (62.4)	
≥5.0	23 (41.8)	62 (37.6)	
Histology differentiation			0.322
Well/moderately	47 (85.5)	131 (79.4)	
Poorly	8 (14.5)	34 (20.6)	

Data are expressed as mean (SD) or percentage. SD, standard deviation; BMI, body mass index; WBC, white blood cell; BUN, blood urea nitrogen; CEA, carcinoembryonic antigen; FPG, fasting plasma glucose; LDH, lactate dehydrogenase; ALP, alkaline phosphatase; ICU, intensive care unit; SOFA, Sequential Organ Failure Assessment; APACHE, Acute Physiology and Chronic Health Evaluation; IQR, 25% and 75% interquartile range; COPD, chronic obstructive pulmonary disease.

**Table 2:** Conditional logistic regression analysis of variables independently associated with postoperative sepsis in CRC patients.

	*β*	HR (95% CI)	*p* value
WBC (×10^9^/L)	0.102	1.107 (0.979–1.252)	0.104
Hemoglobin (g/L)	0.006	1.006 (0.991–1.022)	0.420
LDH (U/L)	0.000	1.000 (0.999–1.002)	0.573
Albumin (g/L)	−0.125	0.883 (0.829–0.940)	<0.001
MPV (fL)	0.754	2.125 (1.677–2.694)	<0.001
PDW (%)	−0.280	0.756 (0.645–0.886)	0.001

HR, hazard ratio; CI, confidence interval; WBC, white blood cell; LDH, lactate dehydrogenase.

**Table 3:** Conditional logistic regression analysis of variables independently associated with ICU mortality in patients with colorectal cancer.

	*β*	HR (95% CI)	*p* value
WBC (×10^9^/L)	−0.165	0.848 (0.646–1.113)	0.234
Hemoglobin (g/L)	0.028	1.029 (1.002–1.057)	0.038
LDH (U/L)	0.001	1.001 (0.999–1.002)	0.409
Albumin (g/L)	−0.124	0.884 (0.805–0.970)	0.010
MPV (fL)	0.610	1.841 (1.369–2.476)	<0.001
PDW (%)	−0.402	0.669 (0.537–0.833)	<0.001

HR, hazard ratio; CI, confidence interval; WBC, white blood cell; LDH, lactate dehydrogenase.

**Table 4:** Conditional logistic regression analysis of variables independently associated with 14-day mortality, 28-day mortality, and 90-day mortality in patients with colorectal cancer.

	*β*	HR (95% CI)	*p* value
14-day mortality			
WBC (×10^9^/L)	0.064	1.066 (0.958–1.186)	0.241
Hemoglobin (g/L)	−0.031	0.969 (0.942–0.997)	0.032
LDH (U/L)	0.002	1.002 (1.000–1.004)	0.045
Albumin (g/L)	−0.185	0.831 (0.750–0.922)	<0.001
MPV (fL)	0.467	1.595 (1.151–2.211)	0.005
PDW (%)	0.109	1.115 (0.890–1.397)	0.344
28-day mortality			
WBC (×10^9^/L)	0.052	1.053 (0.954–1.163)	0.307
Hemoglobin (g/L)	−0.018	0.982 (0.960–1.005)	0.127
LDH (U/L)	0.001	1.001 (1.000–1.003)	0.059
Albumin (g/L)	−0.172	0.842 (0.772–0.917)	<0.001
MPV (fL)	0.316	1.372 (1.042–1.807)	0.024
PDW (%)	0.128	1.136 (0.923–1.399)	0.228
90-day mortality			
WBC (×10^9^/L)	0.103	1.109 (0.985–1.249)	0.088
Hemoglobin (g/L)	0.011	1.011 (0.993–1.029)	0.226
LDH (U/L)	0.001	1.001 (0.999–1.002)	0.307
Albumin (g/L)	−0.143	0.867 (0.809–0.929)	<0.001
MPV (fL)	0.486	1.626 (1.309–2.019)	<0.001
PDW (%)	−0.187	0.830 (0.710–0.970)	0.019

HR, hazard ratio; CI, confidence interval; WBC, white blood cell; LDH, lactate dehydrogenase.

## Data Availability

The clinical data of patients used to support the findings of this study are available from the corresponding author upon request.
